# Cyclin-dependent kinase inhibitor fadraciclib (CYC065) depletes anti-apoptotic protein and synergizes with venetoclax in primary chronic lymphocytic leukemia cells

**DOI:** 10.1038/s41375-022-01553-w

**Published:** 2022-04-05

**Authors:** Rong Chen, Yuling Chen, Ping Xiong, Daniella Zheleva, David Blake, Michael J. Keating, William G. Wierda, William Plunkett

**Affiliations:** 1 Department of Experimental Therapeutics, The University of Texas MD Anderson Cancer Center, Houston, TX USA; 2 Cyclacel Pharmaceuticals Inc, Dundee, UK; 3 Department of Leukemia, The University of Texas MD Anderson Cancer Center, Houston, TX USA

**Keywords:** Drug development, Chronic lymphocytic leukaemia

## Abstract

Fadraciclib (CYC065) is a second-generation aminopurine CDK2/9 inhibitor with increased potency and selectivity toward CDK2 and CDK9 compared to seliciclib (R-roscovitine). In chronic lymphocytic leukemia (CLL), a disease that depends on the over-expression of anti-apoptotic proteins for its survival, inhibition of CDK9 by fadraciclib reduced phosphorylation of the C-terminal domain of RNA polymerase II and blocked transcription in vitro; these actions depleted the intrinsically short-lived anti-apoptotic protein Mcl-1 and induced apoptosis. While the simulated bone marrow and lymph node microenvironments induced Mcl-1 expression and protected CLL cells from apoptosis, these conditions did not prolong the turnover rate of Mcl-1, and fadraciclib efficiently abrogated the protective effect. Further, fadraciclib was synergistic with the Bcl-2 antagonist venetoclax, inducing more profound CLL cell death, especially in samples with 17p deletion. While fadraciclib, venetoclax, and the combination each had distinct kinetics of cell death induction, their activities were reversible, as no additional cell death was induced upon removal of the drugs. The best combination effects were achieved when both drugs were maintained together. Altogether, this study provides a rationale for the clinical development of fadraciclib in CLL, either alone or in combination with a Bcl-2 antagonist.

## Introduction

Chronic lymphocytic leukemia (CLL) is characterized by the gradual accumulation of small, mature lymphocytes, with typical B-cell markers [1]. Several lines of evidence suggest that the survival advantage of CLL lymphocytes is due to the over-expression of anti-apoptotic proteins of the Bcl-2 family [2–4]. These proteins bind to pro-apoptotic proteins to prevent them from disrupting the mitochondrial outer membrane and initiating apoptosis. The mitochondria of the CLL cells are “primed” with death signals, and the cells require the continuous expression of anti-apoptotic proteins to maintain their survival [5, 6].

In this biological context, agents that antagonize or diminish the anti-apoptotic proteins cause the release of pro-death signals that commit cells to apoptosis. This approach has been a focus of new therapeutics in CLL. The key anti-apoptotic proteins that lend a survival advantage to CLL are Bcl-2 and Mcl-1. Inactivation of Mcl-1 function has demonstrated selective killing of CLL cells in vitro [7–11], achieved through an indirect or a direct approach. The indirect approach consists of transient blocking of transcription using inhibitors of cyclin-dependent kinase 9 (CDK9) [7, 8] or staving off protein translation. CDK9 is an integral part of the transcription elongation factor P-TEFb; it phosphorylates the Ser-2 sites in the C-terminal domain (CTD) of RNA polymerase II (Pol II), which is required for transcript elongation. This approach takes advantage of the labile feature of both Mcl-1 mRNA and protein. The Mcl-1 transcript has intrinsic motifs (adenine-uracil-rich elements, or ARE) that target it for rapid turnover by ribonucleases, and the protein contains two dominant PEST sequences that target it for proteasomal degradation [12]. The result of CDK9 inhibition is a rapid and selective loss of Mcl-1 expression, followed by induction of apoptosis. In recent years, using structure-based drug discovery, several direct Mcl-1 inhibitors have been created [13–15]. These compounds demonstrated in vitro efficacy in CLL and are currently under clinical evaluation.

The development of venetoclax (ABT-199), an antagonist of Bcl-2 function, has been a major breakthrough in the treatment of CLL [16]. However, it is now apparent that a substantial fraction of CLL patients do not experience or maintain a response to venetoclax therapy [17]. While there is a low incidence of mutations in the Bcl-2 gene that give rise to venetoclax resistance [18, 19], there are indications that other Bcl-2 family anti-apoptotic proteins may have a larger role in treatment failure [20, 21]. High intrinsic or induced expression of Mcl-1 has been associated with resistance to venetoclax [21]. Thus, we postulate that a combination of venetoclax with approaches that diminish Mcl-1 will block the two major arms of the anti-apoptotic machinery and contribute to a deeper clinical response [22].

The novel CDK inhibitor fadraciclib (CYC065) was designed by optimizing the aminopurine scaffold of seliciclib (R-roscovitine) [23]. It exhibits improved potency and selectivity for CDK2 (IC_50_ 4.5 nM) and CDK9 (IC_50_ 26.2 nM) over the parental compound, as well as high selectivity across the kinome. In acute myeloid leukemia (AML), breast cancer cell lines and primary AML cells, fadraciclib has demonstrated actions that decreased RNA Pol II phosphorylation, reduced Mcl-1 level, and induced apoptosis [23, 24]. The anti-cancer efficacy of fadraciclib has also been observed in vivo in AML xenograft models, in breast cancer models combined with trastuzumab [25] or eribulin [26], in uterine serous carcinoma models combined with taselisib or trastuzumab [27], as well as in MYCN-driven neuroblastoma [28]. CDK2 inhibition by fadraciclib was reported to cause anaphase catastrophe in aneuploid cancers [29]. A phase 1 trial of fadraciclib in patients with advanced solid tumors (NCT02552953) demonstrated signs of clinical benefit with tolerable toxicity, and a phase 1/2 study of oral fadraciclib in patients with advanced solid tumors (NCT04983810) is now underway. The combination of fadraciclib with venetoclax is currently being evaluated in a phase 1/2 study in leukemia or myelodysplastic syndrome (NTC05168904). Here, we present the preclinical evaluation of fadraciclib in CLL, both alone and in combination with venetoclax.

## Methods

Complete methods are presented in the Supplemental Materials.

### Patient samples

Samples from 67 CLL patients and 3 healthy donors were used in this study. The median age of the CLL patients was 64 years (range, 27 to 88 years), with 46 male patients and 21 female patients. Their median white blood cell count was 60,000/μl (range, 15,000 to 339,000/μl). The median lymphocyte percentage was 90% (range, 51–97%). Detailed patient characteristics are summarized in Supplemental Table 1. Approval was obtained from The University of Texas MD Anderson Cancer Center Institutional Review Board for this investigation, and all patients and donors agreed to participate and provided informed consent for use of their cells for in vitro studies.

## Results

### Mechanism of action of fadraciclib in primary CLL cells

**Fig. 1:**
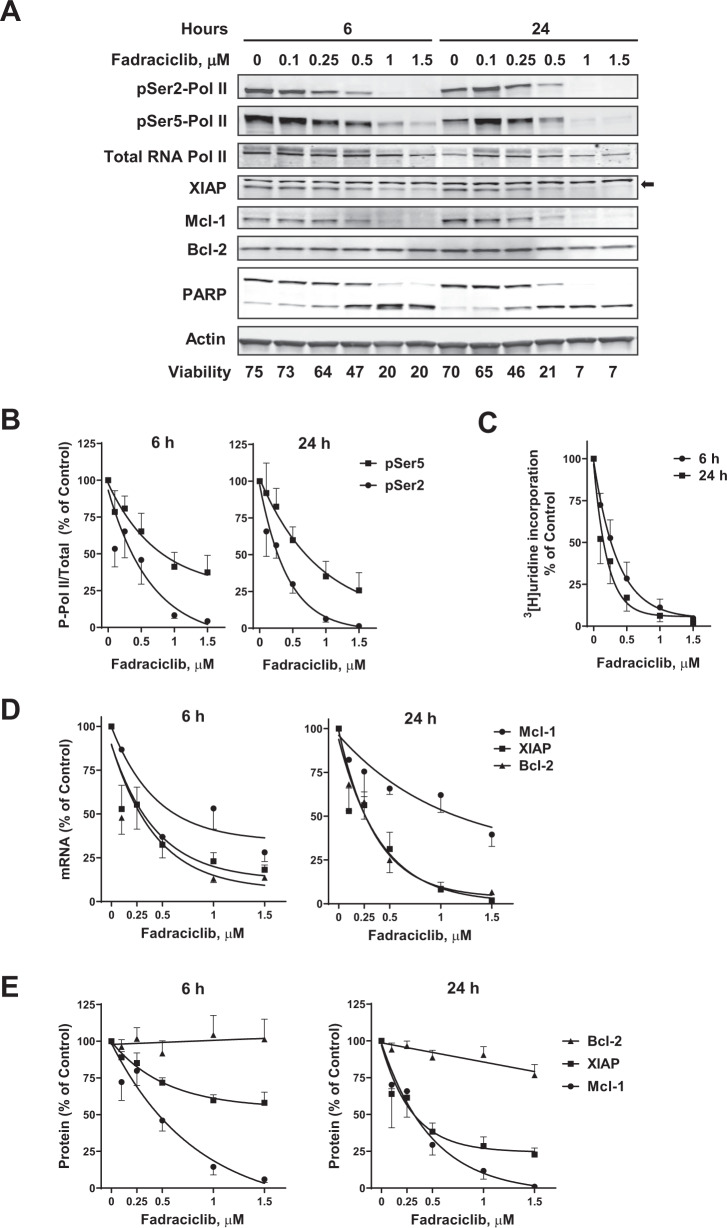
Mechanism of action of fadraciclib in primary CLL cells. **A** Fadraciclib reduced the phosphorylation of RNA Pol II and reduced the anti-apoptotic proteins Mcl-1 and XIAP. CLL cells were incubated with increasing concentrations of fadraciclib for 6 and 24 h, and the phosphorylation of RNA Pol II was analyzed by immunoblotting, using antibodies against the phosphorylated Ser2 or Ser5 sites of the CTD, as well as total Pol II. The major anti-apoptotic proteins Mcl-1, Bcl-2 and XIAP were analyzed with their specific antibodies. PARP was used as an indicator of apoptosis, and actin was used as a loading control. A representative immunoblot of 8 patient samples is shown. Cell viability, measured by annexin V/propidium iodide (PI) and flow cytometry, is shown below the bands. Arrow at right indicates the band for XIAP. **B** Inhibition of phosphorylation of Pol II at Ser2 (●) and Ser5 (■) sites after 6 h (left) and 24 h (right) incubation with fadraciclib. Levels of phosphorylation were quantified from the blots in (**A**), normalized to total Pol II, and then expressed as a percentage of time-matched controls (mean ± standard error of the mean [SE] of 8 individual CLL samples). **C** Inhibition of RNA synthesis by fadraciclib in CLL cells. RNA synthesis was measured by [^3^H]uridine incorporation in 5 CLL samples after 6 (●) and 24 h (■) incubation with increasing concentrations of fadraciclib. Each measurement was performed in triplicate. Data are presented as percentage of time-matched controls (mean ± SE of 5 CLL samples). **D** Fadraciclib reduced mRNA levels of Mcl-1, XIAP, and Bcl-2. mRNA levels of Mcl-1(●), XIAP (■), and Bcl-2 (▲) were measured by real-time RT-PCR, each performed in duplicate, and compared with time-matched controls (mean ± SE of 8 CLL samples). **E** Quantitation of immunoblots of Mcl-1, XIAP, and Bcl-2 from the same samples as described in (**A**). Levels of Mcl-1, XIAP, and Bcl-2 were normalized to actin and expressed as percentage of time-matched controls (mean ± SE of 8 CLL samples).

We first tested whether inhibition of CDK9 by fadraciclib would reduce RNA Pol II phosphorylation in CLL cells and reduce transcription. Indeed, treatment of the primary CLL cells with fadraciclib for 6 or 24 h was associated with a concentration-dependent decrease in phosphorylation of Ser2 and Ser5 of the CTD of Pol II (Fig. 1A). Ser5 of the CTD was phosphorylated by CDK7 to promote transcription initiation [30]. The ratios of pSer2:total Pol II and pSer5:total Pol II were reduced in a concentration-dependent manner (Fig. 1B) that was remarkably consistent between samples. The reduction of Pol II phosphorylation was associated with inhibition of RNA synthesis, measured by tritiated uridine incorporation, which decreased in a concentration-dependent manner after fadraciclib treatment (Fig. 1C). The mean (± standard error of mean, SE) uridine incorporation was reduced to 11.2% ± 5.0% and 6.2% ± 3.6% of controls after 6 and 24 h incubation with 1 μM fadraciclib.

The most sensitive targets of transcription inhibitors are likely to be transcripts and proteins that intrinsically turn over rapidly. Fadraciclib induced a concentration-dependent decrease in the mRNA levels of Mcl-1, Bcl-2, and XIAP (Fig. 1D) at both 6 and 24 h. This was associated with the decreases in the protein levels of Mcl-1 and XIAP in a representative immunoblotting analysis (Fig. 1A). Similar analyses from 8 patient samples were quantified and summarized (Fig. 1E). The rates of decrease in the protein levels were proportional to their intrinsic half-lives, with Mcl-1 being the most labile. There was no significant change in the Bcl-2 protein, consistent with a much longer protein half-life [31].

### Characterization of fadraciclib-induced cell death in primary CLL

**Fig. 2:**
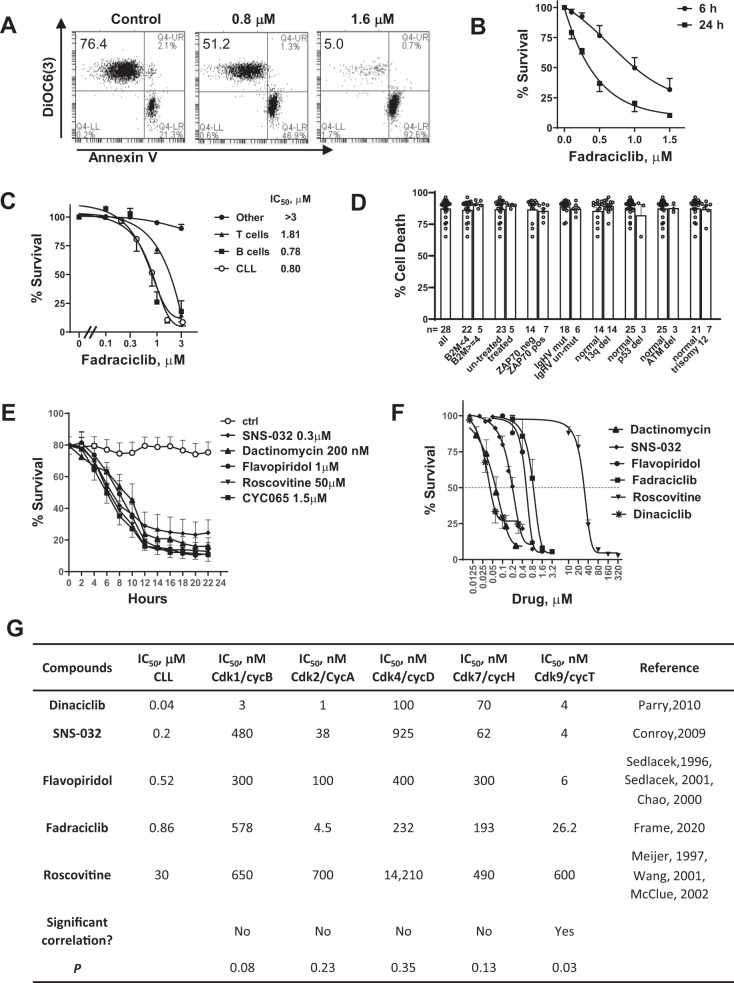
Characterization of fadraciclib-induced CLL cell death. **A** Fadraciclib induced loss of mitochondrial membrane potential and apoptosis in the CLL cells. A representative flow cytometry result of CLL cells incubated with DMSO control or fadraciclib at 0.8 and 1.6 μM for 24 h. The cells were stained with annexin V-Cy5/DiOC6(3) and analyzed by flow cytometry. Cells with intact mitochondrial membrane potential were stained positive for DiOC6(3), and apoptotic cells were stained positive for annexin V. The percentages of live cells (annexin-–negative/DiOC6 + ) were shown in the upper left quadrant. **B** Induction of cell death in CLL cells after 6 h (●) and 24 h (■) exposure to increasing concentrations of fadraciclib. Cell viability was measured by annexin V/PI double staining and quantitated by flow cytometry analysis. Data represent mean ± SE of 5 samples. **C** Comparison of fadraciclib toxicity to CLL cells and to normal B and T cells from healthy donors. Cell survival (mean ± SE) was compared after 24 h incubation with increasing concentrations of fadraciclib in 5 CLL cell samples (○) and normal B cells (■), T cells (▲), and other cells (neither B nor T, ●) from 3 healthy donors. **D** Comparison of cell death induced by fadraciclib in CLL cells from patients with different prognostic characteristics and treatment histories. Cell death induced by 1.5 μM fadraciclib after 24 h incubation was compared in CLL cells from patients with favorable or poor prognostic factors. The number of samples in each group is shown below the columns. None of the comparisons were significantly different (*p* > 0.05). **E** Time course of induction of cell death by the CDK inhibitors and dactinomycin. CLL cells were incubated with approximately 2 × IC_50_ concentrations of each compound for a duration of 22 h, and viability was measured by annexin V/PI double staining every 2 h. Data represent mean viability ± SE in 4 CLL samples. **F** Dose-response curves of the CDK inhibitors and dactinomycin. CLL cells were incubated with various concentrations of each compound for 24 h, and viability was measured by annexin V/PI. Data represent the mean viability ± SE of 5 to 10 samples). **G** Correlation analysis of the IC_50_ values of the CDK inhibitors to induce apoptosis in the CLL cells and their IC_50_ for each CDK. A 2-tailed Spearman analysis showed that the IC_50_ values against CLL cells correlated significantly to their IC_50_ values against CDK9, but not to that of the other CDKs.

Upon removal of Mcl-1 protein, the pro-apoptotic binding partners are freed to disrupt the mitochondrial membrane and induce apoptosis. Depolarization of mitochondrial membranes was detected by the loss of binding to the cationic dye DiOC(6)3, and apoptosis was quantitated by annexin V binding. As shown in Fig. 2A, fadraciclib induced a dose-dependent shifting of cells from the DiOC6(3)-positive, annexin V–negative population to the DiOC6(3)-negative, annexin V–positive population, indicating that loss of mitochondrial membrane potential was associated with the onset of cell death, likely initiated by the reduction in Mcl-1 protein.

CLL cells from 5 patient samples incubated with a range of fadraciclib concentrations (0.1–1.5 μM) for 6 and 24 h demonstrated a time- and concentration-dependent induction of cell death, reaching a maximum at 1.5 μM at 24 h (viability 10.3% ± 0.94% relative to untreated controls, set to 100%) (Fig. 2B). B lymphocytes from healthy donors were similarly sensitive to fadraciclib, whereas the T cells and other peripheral blood mononuclear cells (neither B nor T cells) were less sensitive (Fig. 2C), indicating that the fadraciclib-induced cell death was selective to B cells. However, B cell toxicity may not pose a problem of using fadraciclib in the clinic as the B cell-targeting antibodies such as rituximab, ofatumumab and obinutuzumab were proven to be safe in CLL patients [32]. In addition, fadraciclib was well tolerated in clinical trials in solid tumors either with a 1 h or 4 h infusion [33, 34].

Due to the heterogeneity of CLL patient samples, cellular and molecular markers have been identified that predict CLL disease progression or response to standard therapy containing alkylating agents and purine nucleoside analogs. For example, high beta-2 microglobulin [35], loss of TP53 or ATM loci [36], absence of somatic IGHV gene mutation [37], and high expression of ZAP70 [38] are predictive for aggressive disease or refractoriness to therapy. As sensitivity to fadraciclib varied among CLL samples, we incubated 28 CLL samples with 1.5 μM fadraciclib for 24 h and compared cell death between groups with favorable and poor prognostic characteristics, groups with and without chromosome abnormality or prior treatment (Fig. 2D). The median cell death rate was 87.6%, ranging from 65.2% to 96.3%. None of the comparisons showed a significant difference (*p* > 0.05), indicating that fadraciclib induces apoptosis by a mechanism that may be independent of these prognostic factors.

### Time and dose dependence of responses to agents that inhibit transcription

We compared the time and dose dependence of responses of CLL cells to different agents that inhibit transcription, including the CDK9 inhibitors dinaciclib [39–42], flavopiridol [7, 43], roscovitine [44–46], SNS-032 [8, 47, 48], and fadraciclib, as well as dactinomycin, which interferes with the elongation of growing RNA chains by intercalating into DNA [49]. All the inhibitors followed a similar pattern of time dependence of cell killing, which plateaued at around 12 h (Fig. 2E) with concentrations of 2 × IC_50_. However, potency varied among the drugs (Fig. 2F); dactinomycin was the most potent, and roscovitine was the least. As these compounds differ in their inhibitory profiles against the CDKs, we correlated the IC_50_ of reducing CLL survival and their IC_50_ toward each CDK. A Spearman rank correlation analysis showed that the IC_50_ values for CLL correlated significantly with the IC_50s_ for CDK9, but not with those of any another CDKs (Fig. 2G), supporting that the action of inhibiting CDK9 was likely the major mechanism for CLL killing.

### Fadraciclib overcame microenvironment-mediated protection

**Fig. 3:**
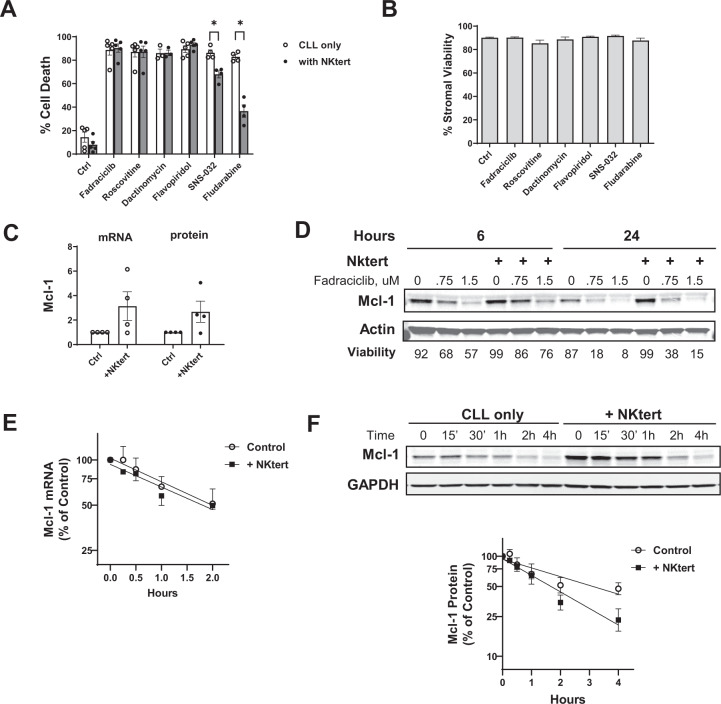
Fadraciclib overcame protection conferred by stroma cells. **A** CLL cells were incubated with approximately 2× IC_50_ concentrations of each compound without (CLL only) or with a layer of StromaNKtert cells (+NKtert) for 24 h, except for fludarabine (48 h). Cell death were measured in the CLL cells by annexin/PI staining and presented as mean ± SE of 5 CLL samples. A paired *t*-test was performed comparing cell death induced by each compound in the “CLL only” group to the “NKtert” group. The differences were not significant except for SNS-032 and fludarabine (noted by the stars above the bars). **B** The compounds used in (**A**) were not toxic to the StromaNKtert cells. The StromaNKtert cells were exposed to the same concentrations of the compounds at the same conditions as in (**A**), and the viability of the stroma cells was measured by annexin/PI staining. Data represent viability (mean ± SD) of measurements performed in triplicates. **C** Co-incubation of the CLL cells with the stroma cells induced Mcl-1 mRNA and protein expression. The CLL cells were incubated with or without the stroma layer for 24 h. The mRNA level of Mcl-1 was measured by real-time RT-PCR, and the protein level was measured by immunoblotting; each was expressed as the ratio to the levels of the controls in CLL cells cultured alone (mean ± SE of 4 CLL samples). **D** CLL cells reduced Mcl-1 level in the presence of StromaNKtert cells. CLL cells were incubated in the absence or presence of stroma cells at increasing concentrations of fadraciclib for 6 and 24 h. Mcl-1 levels were determined by immunoblotting. One immunoblot that is representative of those obtained from 3 patient samples is shown, and cell viabilities are displayed below the image. **E** Mcl-1 mRNA half-life in the presence or absence of stroma cells. CLL cells were incubated with 5 μg/ml dactinomycin in the presence or absence of stroma cells. The cells were collected at 0, 0.25, 0.5, 1, 2, and 4 h, and the Mcl-1 mRNA level was measured by real-time RT-PCR and plotted as percentage of Mcl-1 level over the controls (mean ± SE of 4 samples). **F** The half-life of Mcl-1 protein in the absence and presence of the stroma cells. CLL cells were incubated with 50 μg/ml cycloheximide in the presence and absence of stroma cells. Cell pellets were collected at indicated times, and Mcl-1 levels were measured by immunoblotting. In all samples, Z-VAD-FMK (50 μM) was added to prevent loss of Mcl-1 due to caspase cleavage. A representative immunoblot from 5 experiments is shown, and the protein levels were quantified and plotted as percentage of Mcl-1 level/controls (mean ± SE of 5 CLL samples).

The in vivo microenvironment provides protective signaling that is harnessed by CLL to promote cancer cell proliferation and survival [50]. To mimic the in vivo bone marrow microenvironment, we incubated the CLL cells on top of a layer of StromaNKtert cells [51]. The StromaNKtert cells clearly protected the CLL cells from spontaneous apoptosis, reducing the average cell death in the control cells from 14.40% to 7.95% (*n* = 5) (Fig. 3A). However, StromaNKtert cells did not protect CLL cells from apoptosis induced by fadraciclib, roscovitine, dactinomycin, or flavopiridol at 2 × IC_50_ concentrations, but did attenuate the effects of SNS-032 and the nucleoside analog fludarabine. None of the compounds affected the viability of StromaNKtert cells (Fig. 3B). Incubation with the StromaNKtert cells increased Mcl-1 mRNA and protein levels by an average of 3.1-fold and 2.7-fold, respectively, measured by real-time reverse transcription-polymerase chain reaction and immunoblotting analysis (Fig. 3C, D). This increase was effectively abrogated by fadraciclib (Fig. 3D). Since the efficiency of the transcription and translation inhibitors is dependent on the rapid turnover rate of Mcl-1, we measured the half-lives of Mcl-1 mRNA and protein co-cultured with the stroma cells. As shown in Fig. 3E, F, the half-lives of Mcl-1 mRNA was 2.0 h and 1.8 h for control and StromaNKtert co-culture conditions, and the Mcl-1 protein half-lives were 3.1 h and 1.7 h for control and co-culture conditions, suggesting that the stroma cells did not stabilize the Mcl-1 mRNA nor its protein and thus did not prevent fadraciclib from diminishing Mcl-1 protein.

**Fig. 4:**
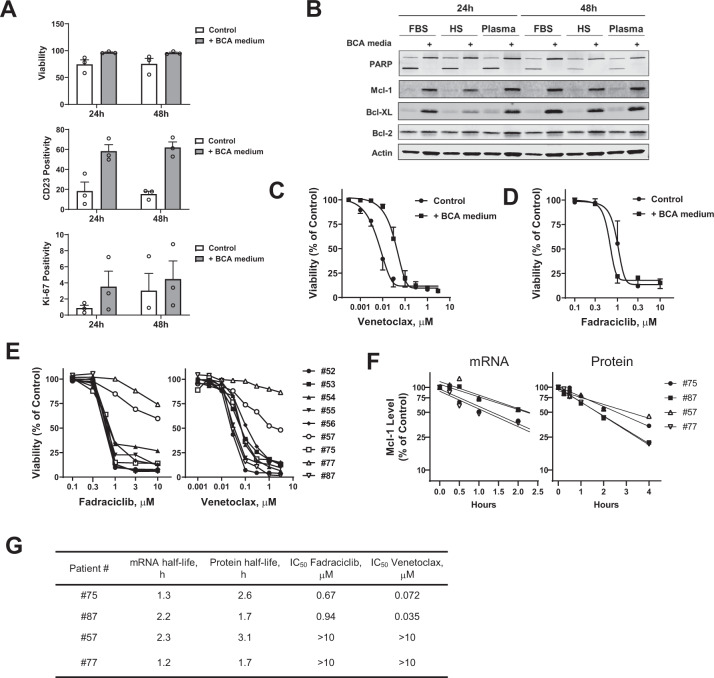
Fadraciclib overcame lymph node microenvironment–mediated protection. **A** B-cell activation (BCA) medium activates CLL cells and promotes CLL survival and proliferation. CLL cells were incubated without or with BCA medium for 24 and 48 h. Cell viability, CD23 positivity, and Ki-67 positivity were measured by flow cytometry (mean ± SE of 3 samples). **B** The BCA medium induced the pro-survival Bcl-2 family proteins and reduced PARP cleavage in the CLL cells. The CLL cells were incubated for 24 and 48 h in RPMI-1640 medium supplemented with either fetal bovine serum (FBS), human AB type serum (HS), or autologous patient plasma with or without BCA medium. Expression of the pro-survival Bcl-2 family proteins and PARP cleavage are shown in a representative immunoblot. **C** The comparison of dose response to venetoclax in CLL cells cultured in the absence (control) or presence of B-cell activation (+BCA medium) (mean ± SE of 3 samples). **D** The comparison of dose response to fadraciclib in CLL cells cultured in the absence (control) or presence of B-cell activation (+BCA medium) (mean ± SE of 3 samples). **E** Heterogenous responses of the CLL cells to fadraciclib and venetoclax. CLL cells were incubated with increasing concentrations of fadraciclib or venetoclax for 24 h, and cell viability was measured by annexin V/PI and normalized to the untreated controls. **F** Measurement of Mcl-1 mRNA and protein half-lives in 4 representative CLL samples with differing sensitivity to fadraciclib and venetoclax. The results are summarized in (**G**) together with the IC_50_ of each sample toward fadraciclib and venetoclax.

To simulate the effect of the lymph node microenvironment [52] on fadraciclib actions, we supplemented the CLL cells with B-cell activation (BCA) medium consisting of anti-CD40 monoclonal antibody, IL-4, and anti-IgM to mimic the T-cell stimulation and engagement of B-cell receptor signaling. This condition activated the CLL B cells, shown by the increased expression of CD23 on the CLL surface (Fig. 4A). The use of BCA medium was also associated with enhanced CLL viability. Similarly, an increase in Ki-67 positivity indicated induction of CLL proliferation by BCA medium (Fig. 4A). An immunoblot showed substantial induction of Mcl-1 and Bcl-XL expression but little change in Bcl-2 associated with the BCA medium (Fig. 4B). A reduction in PARP cleavage in BCA medium was consistent with the increase in CLL survival. CLL cells under this protective condition were less sensitive to the Bcl-2 inhibitor venetoclax, as shown by an averaged 6-fold increase in the mean IC_50_ from 0.007 μM to 0.042 μM (Fig. 4C). However, this condition did not protect the CLL cells from fadraciclib-induced apoptosis; on the contrary, the IC_50_ of fadraciclib was lower in BCA medium (0.7 μM) compared to the controls (1.1 μM) (Fig. 4D). We found that the sensitivity to fadraciclib and venetoclax varied between the CLL samples (Fig. 4E), and identified two samples (#57 and #77) that were relatively resistant to both compounds. To test if the Mcl-1 half-life affected drug sensitivity, we compared the Mcl-1 mRNA and protein half-lives in #57 and #77 to those of two samples that were more sensitive to both compounds (#75 and #87; Fig. 4F). As summarized in Fig. 4G, there was no correlation between Mcl-1 half-life and sensitivity to fadraciclib or venetoclax. All these experiments were carried out in CLL cells incubated in the BCA medium. The average half-lives of Mcl-1 mRNA and proteins measured in the BCA medium were 1.8 h and 2.3 h, respectively, which were similar to or even shorter than those measured previously without the BCA medium (2.0 h and 3.1 h, respectively; Fig. 3E, F), indicating that the lymph node microenvironment did not slow down the turn-over rate of Mcl-1 mRNA and protein.

Since the BCA medium provided excellent protection for the CLL cells in vitro and represents a microenvironment that is more relevant to the clinical challenge where venetoclax seems to be less effective in the lymph nodes, all our later experiments were performed in CLL cells cultured in the BCA medium.

### Synergistic combinations of fadraciclib and venetoclax

**Fig. 5:**
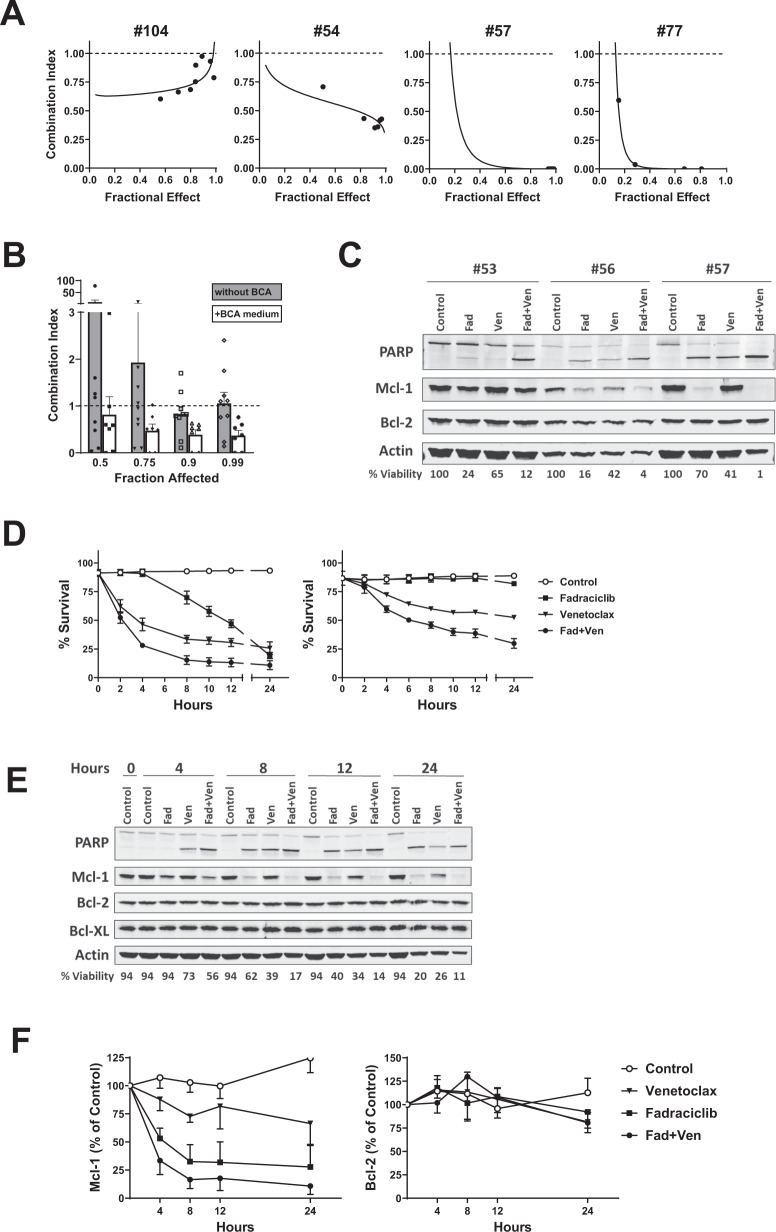
Synergistic combinations of fadraciclib and venetoclax. **A** Representative median-effect plots of the combination of fadraciclib and venetoclax in representative CLL samples cultured without BCA medium (#104) and with BCA medium (#54) and 2 samples with a high percentage of 17p deletion (#57, #77) cultured in BCA medium. **B** Comparison of the combination index for the combination of fadraciclib and venetoclax in CLL cells cultured without (gray column, 9 samples) or with BCA medium (white column, 7 samples). Data are presented as mean ± SE. **C** Representative immunoblotting of the combination of fadraciclib (Fad) and venetoclax (Ven) showing PARP cleavage and Mcl-1 and Bcl-2 protein levels. The CLL cells were incubated with 1 X IC_50_ concentrations of each drug (measured previously for each sample) for 24 h. The percentage of cell viability relative to controls is shown below the images. **D** Time course of loss of CLL viability induced by fadraciclib, venetoclax, and the combination. CLL cells were incubated with the average IC_50_ concentrations of fadraciclib (0.8 μM), venetoclax (0.07 μM), and the combination. Cell viability was measured at indicated times up to 24 h. Left: representative of 3 CLL samples without 17p deletion. Right: representative 2 CLL samples with 17p deletion (67% and 95% del17p). **E** Immunoblot showing the time effect of fadraciclib, venetoclax, and the combination in the samples described in **D**. **F** The levels of Mcl-1 (left) and Bcl-2 (right) were quantified in the blots and plotted as percentage of 0-h controls (mean ± SE of 3 samples).

We evaluated the combination effect of fadraciclib and venetoclax in CLL cells in different conditions: cultured in regular CLL culture medium (RPMI + 10% autologous plasma, *n* = 9, represented by sample #104) and in BCA medium (*n* = 3, represented by #54), and in samples with 17p deletion cultured in BCA medium (*n* = 4, represented by #57 and #77) (Fig. 5A). The combination was synergistic (combination index < 1) in all conditions. We found that the synergistic effect was stronger in samples cultured in BCA medium, demonstrated by a much lower combination index (Fig. 5B). Among the 7 samples cultured with BCA medium, 4 had del17p, and 2 of them (#57, #77) had been highly resistant to fadraciclib and venetoclax as single agents (IC_50_ > 10 μM). However, both samples were exceptionally sensitive to the combination, represented by the extremely low combination index in the median-effect graphs in Fig. 5A. An immunoblot of 3 CLL samples showed that fadraciclib reduced Mcl-1 levels but spared Bcl-2, whereas venetoclax did not have much effect on Mcl-1. Compared with the single agents, the combination caused a greater reduction of Mcl-1, more extensive cleavage of PARP, and greater reduction of CLL survival (Fig. 5C). A dose reduction index analysis [53, 54] showed that the combination greatly reduced the concentrations of fadraciclib and venetoclax needed to reach a fraction of 0.5, 0.75, 0.9, and 0.99 CLL killing compared to the drugs used alone (Table 1).

**Table 1:** Synergistic combinations of fadraciclib and venetoclax.

Combination Index										
Fraction Affected	no BCA medium			with BCA medium			del17p with BCA medium			
	#52	#54	#55	#52	#54	#55	#53	#56	#57	#77
0.5	4.1	0.1	0.04	3.0	0.6	1.0	0.5	0.6	0.03	0.003
0.75	1.8	0.1	0.1	1.0	0.5	0.8	0.5	0.5	0.003	0.0003
0.9	0.8	0.1	0.2	0.6	0.4	0.6	0.5	0.5	2.4E-04	5.0E-05
0.99	0.1	0.2	1.8	0.5	0.3	0.4	0.6	0.8	1.3E-06	2.0E-07
**Dose Reduction Index**^**a**^ **for Fadraciclib**										
Fraction Affected	**no BCA medium**			**with BCA medium**			**del17p with BCA medium**			
	#52	#54	#55	#52	#54	#55	#53	#56	#57	#77
0.5	0.4	11.3	27.0	0.4	3.3	1.4	2.5	3.9	6374.2	660.3
0.75	0.8	12.6	10.7	2.0	3.9	2.0	2.2	3.0	1.9E + 05	2.8E + 04
0.9	1.7	14.1	4.3	10.0	4.7	2.8	2.0	2.4	5.8E + 06	1.2E + 06
0.99	9.1	18.0	0.6	326.3	7.0	3.5	1.7	1.4	9.7E + 09	4.0E + 09
**Dose Reduction Index**^**a**^ **for Venetoclax**										
Fraction Affected	**no BCA medium**			**with BCA medium**			**del17p with BCA medium**			
	#52	#54	#55	#52	#54	#55	#53	#56	#57	#77
0.5	0.8	122.7	2192.0	1.9	3.5	3.4	10.4	2.9	34.0	965.2
0.75	1.9	58.8	1395.6	1.9	4.0	3.7	26.5	5.3	376.2	4492.0
0.9	4.5	28.1	888.5	1.9	4.5	4.0	67.7	9.4	4137.4	2.1E + 04
0.99	29.4	5.6	331.6	1.9	5.9	4.3	523.7	33.9	7.9E + 05	6.0E + 05

^**a**^The dose reduction index is a measure of how many fold the dose of each drug in a synergistic combination may be reduced, at a given effect level, compared with the doses of each drug alone.

A detailed time course analysis of cell death induced by fadraciclib, venetoclax, and the combination showed distinct kinetics between the treatments. Fadraciclib at the average IC_50_ concentration (0.8 μM) reduced CLL viability gradually over the 24 h period (Fig. 5D). This differed from the previous results, where cell death plateaued at 10–12 h (Fig. 2E), indicating that the BCA medium delayed the cell death from fadraciclib. On the contrary, venetoclax (0.07 μM) initiated cell death quickly, within a few hours, and then cell death stabilized after 6–8 h. The synergistic effect of the combination was apparent as early as 2 h, and cell death followed a pattern similar to that of venetoclax. The 2 samples with 17p deletion (Fig. 5D, right panel, 67% and 95% del17p respectively) were less sensitive to the single drug, but were sensitive to the combination. Representative immunoblots (Fig. 5E) and quantitations (Fig. 5F) demonstrated gradual reduction of Mcl-1 within 8 h by fadraciclib. Venetoclax reduced Mcl-1 only marginally, likely due to cleavage of Mcl-1 by the activated caspase, while the combination caused greater Mcl-1 reduction. The levels of Bcl-2 remained stable over the time course.

### Cell death induced by fadraciclib and venetoclax was reversible

**Fig. 6:**
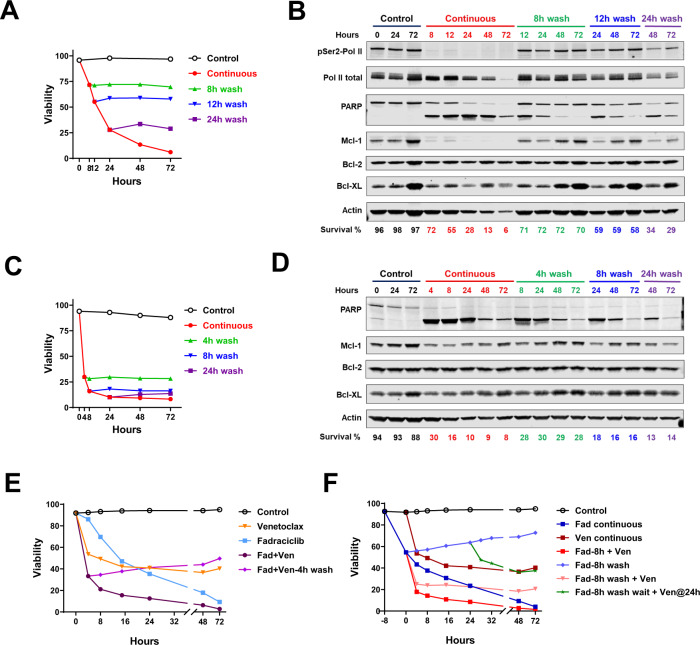
Cell death induced by fadraciclib and venetoclax was reversible. **A** CLL cells were incubated with fadraciclib (0.8 μM) for 8, 12, and 24 h before they were washed and re-incubated with drug-free medium, and cell viability was measured at the indicated times up to 72 h. One representative graph from 2 samples is shown. **B** Representative immunoblot from 2 samples showing recovery of RNA Pol II phosphorylation, Mcl-1 level, and PARP cleavage after wash. **C** CLL cells were incubated with venetoclax (0.07 μM) for 4, 8, and 24 h before they were washed and re-incubated with drug-free medium, and cell viability was measured at the indicated times up to 72 h. One representative graph from 2 samples is shown. **D** Representative immunoblot of the samples collected in C from 2 samples. **E** Reversible cell death induced by the combination of fadraciclib and venetoclax. A representative time course of viability in CLL cells incubated with IC_50_ concentrations of fadraciclib (0.87 μM), venetoclax (0.05 μM), and the combination for the indicated times; the combination sample (purple) was washed at 4 h before incubation in the drug-free medium. **F** Effects of tandem administration of fadraciclib and venetoclax on different schedules. CLL cells were incubated with IC_50_ concentration of fadraciclib. After 8 h, the cells either continued in fadraciclib till 72 h (Fad continuous, dark blue) or received the addition of the IC_50_ concentration of venetoclax (Fad-8h + Ven, red). The rest of the cells were washed at 8 h, then re-incubated in drug-free medium (Fad-8h wash, blue) or with venetoclax (Fad-8h wash + Ven, pink), or wait until 24 h before adding venetoclax (Fad-8h wash wait + Ven@24 h, green). Cell viability was measured at indicated times. The cells incubated in drug-free medium (control) or venetoclax (venetoclax continuous, maroon) served as controls. A representative plot from 3 samples is shown.

Our previous experience showed that the cell death induced by CDK9 inhibitors was reversible [8]. To test if the same held for fadraciclib, we incubated the CLL cells with fadraciclib for 8, 12, and 24 h in the BCA medium, washed the drug off, and then continued incubating the cells in drug-free medium until 72 h. Whereas there was a gradual decrease in viability with continuous fadraciclib exposure, there was no additional cell death upon drug removal (Fig. 6A). The representative immunoblots showed recovery of Pol II phosphorylation as soon as 4 h, and recovery of Mcl-1 protein and a reduction of PARP cleavage (Fig. 6B). There was an increase in Mcl-1 and Bcl-XL over prolonged incubation with BCA medium, consistent with our previous observation. Similarly, the cell death induced by venetoclax (Fig. 6C, D) as well as the combination (Fig. 6E) was also reversible.

We continued to investigate the effects of tandem drug administration on various schedules. When we incubated the CLL cells with fadraciclib for 8 h before adding venetoclax, the addition of venetoclax greatly enhanced cell death (Fig. 6F, red line). However, the killing was reduced if the fadraciclib was washed off before the addition of venetoclax (pink line). Furthermore, if we waited until 24 h following washout of fadraciclib before the administration of venetoclax (green line), the cell death was even less. These results demonstrate that the best combination effect occurred when both drugs were present at the same time.

## Discussion

In this study, we investigated the action of the novel CDK inhibitor fadraciclib, alone and in combination with a Bcl-2 antagonist, in primary CLL cells. Through inhibition of CDK9, fadraciclib reduced RNA Pol II phosphorylation at the C-terminal domain and blocked transcription. This diminished the short-lived Mcl-1 mRNA and protein and induced apoptosis in the CLL cells. In addition, although the simulated CLL microenvironment induced Mcl-1 expression and protected CLL cells from apoptosis, fadraciclib efficiently abrogated the protective effect. Furthermore, fadraciclib was synergistic with the Bcl-2 antagonist venetoclax, inducing more profound CLL cell death, especially in samples with deletion of 17p.

Cyclin-dependent kinases are essential to promote uncontrolled proliferation, an important hallmark of cancer. In the past decades, a great amount of effort has been directed toward developing inhibitors of CDK, resulting in the success of CDK4/6 inhibitors (palbociclib [55], ribociclib [56], and abemaciclib [57]) for the treatment of advanced breast cancer. In addition to proliferation, CDKs are involved in the control of transcription, including CDK7, 8, 9, 12, and 13. Among those, CDK9 together with cyclin T form the positive transcription factor P-TEFb, which phosphorylates the CTD of RNA Pol II to promote transcription elongation [58]. A series of CDK9 inhibitors have been developed and actively tested in vitro, in animal models, and in clinical trials. We showed that the IC_50_ values of various CDK9 inhibitors against CLL samples correlated with the IC_50_ against CDK9, but not against other CDKs, further supporting that CDK9 inhibition is a common mechanism of action for these compounds. The anti-cancer activity of CDK9 inhibitors relies largely on the rapid turnover rate of the oncoprotein target, as well as the critical dependence of the cancer cells on the sustained expression of the oncoprotein for survival. In addition to hematological malignancies where Mcl-1 is the major target, Myc-driven diseases such as neuroblastoma are a trending area for the development of CDK9 inhibitors [28, 59], with a few compounds being actively tested in the clinic.

Bcl-2 and Mcl-1 are key survival proteins that sustain the CLL cells. Inhibiting Bcl-2 alone, using venetoclax, was efficient in inducing apoptosis in the CLL cells. However, venetoclax has little effect on Mcl-1. Venetoclax is known to be more effective in removing the CLL cells in the blood and bone marrow than in the lymph nodes, which are associated with a low rate of complete remission in the clinic [17, 60]. The success of combining venetoclax with a BCR signaling inhibitor is an example of complementing the action of venetoclax with agents targeting the lymph node microenvironment [61, 62]. This microenvironment induced major anti-apoptotic proteins such as Mcl-1 and Bcl-XL, leading to resistance to venetoclax. This finding opens possibilities for rationally combining venetoclax with other targeted agents to circumvent resistance, especially agents that antagonize Mcl-1. On the other hand, fadraciclib reduced Mcl-1 expression by transcription inhibition but spared Bcl-2, owing to the long half-life of Bcl-2 protein. Thus, the combination of fadraciclib and venetoclax targeted the parallel arms of apoptosis control and killed the CLL cells synergistically (Fig. 5). This synergy was even stronger in CLL cells cultured in a condition mimicking the lymph node microenvironment (Fig. 5B), likely because the cells became more dependent on Mcl-1.

Patients bearing 17p deletion remain a challenge in CLL therapy. Deletion of the TP53 gene in this region and simultaneous mutation on the other allele disrupt p53 function and may contribute substantially to its pathogenesis. Studies showed that TCL1 transgenic mice with p53 gene mutation exhibited higher survival capacity and were more drug-resistant than the wild-type mice. In addition, 17p deletion may decrease miR-15a/miR-16-1 and increase Mcl-1 expression [63]. TP53 loss also may increase the threshold for BAX/BAK activation and was found to reduce sensitivity to venetoclax and to an Mcl-1 inhibitor [64]. A recent study revealed an epigenetic regulatory mechanism involving a new tumor suppressor gene, *PHF23*, which may contribute to the pathology of 17p-deleted cancers [65]. In our study, the 2 CLL samples with a high percentage of 17p deletion (90% and 86% for #57 and #77, respectively) demonstrated resistance to both fadraciclib and venetoclax. Sample #57 carries a 22-base-pair insertion in exon 8 of the remaining allele, leading to frameshift of the coding region. There was no information regarding the p53 mutation status for sample #77. Interestingly, in both samples, there was remarkable synergy of the combination of fadraciclib and venetoclax, shown by combination index values far below 0.1. This synergy was accompanied by high dose reduction index values, indicating that the combination greatly reduced the dose for individual drugs to reach a specific killing. Thus, the combination strategy has great potential to overcome drug resistance that was associated with 17p deletion.

Although the mechanism of action and anti-cancer efficacy of the CDK9 inhibitors have been validated both in vitro and in animal models, their clinical outcomes have not been satisfactory. Rapid plasma clearance likely contributed to their clinical failure. Our data showed that this group of compounds shared a similar time course of cell death induction. This time dependence was also observed with the transcription inhibitor dactinomycin, indicating a common mechanism of action. We also showed that cell death induced by fadraciclib was reversible. RNA Pol II phosphorylation and Mcl-1 level were both recovered upon washing away of the drug, accompanied by a lack of additional cell killing. This observation emphasizes the need to maintain an active plasma concentration of CDK9 inhibitor for over 10–12 h in the clinic. In addition, we showed that the best combination effect was achieved when the both fadraciclib and venetoclax were present simultaneously, compared to sequential administration (Fig. 6F). This information is a valuable to guide clinical trial design for testing fadraciclib in CLL. Pharmacokinetic studies of 1 h infusion of fadraciclib in solid tumors showed a C_max_ of 6.2 μM and a half-life of up to 3.5 h at 213 mg dose. The in vitro active concentration of fadraciclib (0.8 μM IC_50_) is attainable and well maintained in vivo [34]. This is a favorable pharmacokinetic feature of fadraciclib. A recent development of an oral formulation of fadraciclib demonstrated a comparable pharmacokinetic profile to that of 1 h intravenous infusion and will be used in an amended clinical trial in CLL.

In summary, our investigations of the mechanism of action of the novel CDK2/9 inhibitor fadraciclib in primary CLL cells confirmed that inhibition of CDK9 mediated transcription reduced Mcl-1 level and induced apoptosis in the CLL cells. Fadraciclib overcame microenvironment-mediated protection and combined synergistically with venetoclax. The cell killing by fadraciclib, venetoclax, and the combination was reversible. The best combination effect was achieved when both drugs were present simultaneously. Thus, this study provides a rationale for the clinical development of fadraciclib in CLL, either alone or in combination with a Bcl-2 antagonist.

## Supplementary information

Supplemental Table 1,materials and methods
