# Supplementary material for: Cyclin-dependent kinase inhibitor fadraciclib (CYC065) depletes anti-apoptotic protein and synergizes with venetoclax in primary chronic lymphocytic leukemia cells
Source: Leukemia. 2022 Apr 5;36(6):1596–608. doi: 10.1038/s41375-022-01553-w (PMC9162916; doi:10.1038/s41375-022-01553-w)
Supplement: Supplementary file 1 — Supplemental Table 1,materials and methods [file 41375_2022_1553_MOESM1_ESM.docx]

**Supplemental Table 1: Characteristics of the CLL patient samples.**

| Pt# | Age, y | Sex | WBC | B2M^a^ | Treatment history^b^ | % 13q del^c^ | % p53 del^c^ | % ATM del^c^ | % Tri12^c^ | ZAP-70^d^ | IgHV mut^e^ |
| --- | --- | --- | --- | --- | --- | --- | --- | --- | --- | --- | --- |
| 1 | 79 | F | 39 | 3.7 | U |  |  |  | 73 | pos | U |
| 2 | 80 | F | 21 | 2.1 | U | NA | NA | NA | NA | NA | M |
| 3 | 66 | M | 25 | 2.7 | U | 62 |  | 52 |  | neg | M |
| 4 | 61 | M | 59 | 3.2 | U | NA | NA | NA | NA | neg | U |
| 5 | 46 | M | 41 |  | U |  |  |  |  | neg | U |
| 6 | 45 | M | 139 | 2.4 | U | 10 |  |  |  | neg | M |
| 7 | 75 | M | 151 | 3.3 | U | 97 |  | 93 |  | pos | U |
| 8 | 72 | M | 75 | 3.2 | T | 52 | 12 | 25 |  | NA | M |
| 9 | 60 | F | 120 | 3.2 | U | 23 |  |  |  | neg | M |
| 10 | 55 | F | 98 | 2.4 | U |  |  |  |  | neg | M |
| 11 | 45 | M | 59 | 2.2 | U |  |  |  | 77 | neg | U |
| 12 | 82 | M | 60 |  | T | 98 | 98 |  |  | NA | NA |
| 13 | 62 | M | 43 | 2.4 | U |  |  |  |  | neg | U |
| 14 | 64 | F | 29 | 3.5 | U |  | 18 |  |  | neg | NA |
| 15 | 51 | M | 339 | 3.9 | U | 19 |  |  |  | neg | M |
| 16 | 52 | M | 65 | 2.5 | U |  |  |  |  | pos | U |
| 17 | 69 | M | 57 | 3.9 | U |  |  |  | 35 | pos | M |
| 18 | 47 | M | 39 | 2.9 | U |  |  |  |  | NA | NA |
| 19 | 62 | F | 36 | 1.5 | U | NA | NA | NA | NA | neg | M |
| 20 | 79 | F | 46 | 3.7 | U |  |  |  | 73 | NA | U |
| 21 | 66 | M | 27 | 2.6 | U |  |  |  |  | NA | M |
| 22 | 64 | F | 50 | 3.9 | T | 76 |  |  |  | pos | U |
| 23 | 68 | M | 41 | 2.1 | U | 92 |  |  | 69 | neg | M |
| 24 | 53 | M | 35 | 1.8 | U |  |  |  |  | neg | M |
| 25 | 82 | M | 80 | 3.6 | T | 99 |  |  |  | NA | M |
| 30 | 64 | F | 32 | 3 | U | 85 |  |  |  | NA | NA |
| 31 | 58 | M | 21 | 2.4 | U |  |  |  | 39 | neg | NA |
| 32 | 62 | F | 43 | 1.7 | U | 82 |  |  |  | neg | M |
| 33 | 59 | M | 174 | 2.9 | U |  |  |  | 10 | pos | U |
| 34 | 65 | F | 33 | 2.2 | T | 75 |  |  |  | pos | U |
| 35 | 74 | F | 74 | 1.9 | U | NA | NA | NA | NA | NA | NA |
| 40 | 36 | F | 70 | 1.8 | U | 9 |  |  |  | NA | NA |
| 42 | 70 | F | 20 | 2.5 | U | NA | NA | NA | NA | neg | M |
| 43 | 53 | M | 44 | 1.6 | U | 30 |  |  |  | pos | U |
| 44 | 67 | M | 37 | 4.5 | U | 66 |  |  |  | NA | NA |
| 45 | 61 | M | 87 | 2.4 | T |  |  |  | 66 | pos | M |
| 46 | 83 | M | 24 | 2.1 | U |  |  |  | 23 | neg | M |
| 47 | 82 | F | 95 | 2 | U | NA | NA | NA | NA | NA | NA |
| 48 | 55 | M | 56 | 1.5 | T | 84 |  |  |  | neg | M |
| 49 | 60 | M | 15 | 2.6 | T |  |  |  |  | pos | NA |
| 52 | 60 | M | 157 | 1.8 | U |  |  |  |  | neg | M |
| 53 | 50 | M | 74 | 2.6 | U |  | 95 |  |  | pos | NA |
| 54 | 72 | M | 51 | 2.9 | U | 37 |  |  | 21 | neg | M |
| 55 | 69 | M | 170 | 4.9 | U | 63 |  | 61 |  | pos | U |
| 56 | 67 | M | 210 | 2.5 | U |  | 54 |  | 44 | pos | U |
| 57 | 60 | M | 103 | 6.5 | U | 93 | 91 |  |  | neg | U |
| 59 | 64 | M | 91 | 3.5 | U | 95 | 95 |  |  | neg | NA |
| 60 | 48 | M | 40 | 1.7 | U |  |  |  |  | pos | M |
| 61 | 64 | M | 60 | 4.9 | T | 94 | 95 | 95 |  | pos | U |
| 62 | 60 | M | 57 | 2.4 | U |  |  |  |  | pos | U |
| 63 | 63 | M | 81 | 3.5 | U | 78 |  |  |  | pos | U |
| 65 | 56 | F | 100 | 2.3 | U |  |  |  |  | neg | M |
| 66 | 66 | M | 90 | 3.3 | U | 46 |  |  |  | pos | U |
| 67 | 69 | M | 253 | 6.4 | U | 51 |  | 63 |  | pos | U |
| 75 | 69 | M | 87 | 3 | U |  |  |  |  | pos | NA |
| 77 | 64 | M | 102 | 3.6 | U |  | 86 |  |  | pos | U |
| 81 | 66 | F | 89 | 2.1 | U |  |  |  |  | neg | M |
| 86 | 60 | M | 240 | 3.9 | U | 95 |  |  |  | NA | M |
| 87 | 77 | M | 170 | 8.9 | U |  |  |  |  | pos | M |
| 101 | 88 | M | 46 | 4.8 | T | 33 |  |  | 65 | neg | M |
| 102 | 64 | F | 127 | 2.7 | U | 89 |  |  |  | neg | M |
| 103 | 84 | F | 54 | 7.3 | U | 88 |  |  |  | NA | M |
| 104 | 77 | M | 77 | 2.9 | U |  |  |  | 74 | neg | M |
| 105 | 27 | M | 55 | 6.7 | U |  |  |  |  | pos | U |
| 106 | 64 | F | 106 | 5.1 | T |  | 41 |  | 59 | pos | M |
| 107 | 51 | M | 24 | 1.9 | U |  |  |  |  | pos | M |

NA: information not available

a: B2M: beta-2-microglobulin, mg/L

b: T: treated; U: untreated

c: Detected using fluorescence in situ hybridization of bone marrow cells using fluorescent probes designed for simultaneous detection of the ATM gene (11q22.3), TP53 gene (17p13.1), D12Z3 (centromeric region of chromosome 12), and the D13S319 locus (13q14.3). A total of 200 interphases were analyzed for each probe. Numbers represent percentage of cells that are abnormal in at least one of the alleles of the gene loci. The normal cutoff is 5.1% for deletion of an ATM gene; 4.5% for deletion of a TP53 gene; 3.5% for deletion of the D13S319 locus; 2.4% for trisomy 12.

d: ZAP-70 expression was detected with either fluorescence in situ hybridization or flow cytometry. Sample with greater than 20% of CLL cells expressing ZAP-70 is considered ZAP-70 positive.

e: IgHV gene with less than 98% homology with the corresponding germ-line gene was considered mutated (M). U: unmutated.

#### Supplemental Materials and Methods:

#### Cell line:

The human mesenchymal cell line StromaNKtert was provided by Dr. Jan Burger from our institution and was originally purchased from the RIKEN Cell Bank (Tsukuba, Ibaraki, Japan). The cell line was authenticated by short tandem repeat DNA fingerprinting at the Cytogenetics and Cell Authentication Core in the Department of Genetics at MD Anderson Cancer Center. The cells were maintained in 1640 medium (Sigma-Aldrich, St. Louis, MO) supplemented with 10% fetal bovine serum. For co-culture experiments, the StromaNKtert cells were seeded onto the 6-well plates at 1 × 10^5^ cells/well the day before co-culturing. CLL cells were added onto the stromal layers at a ratio of 100 to 1 (CLL: StromaNKtert).

#### Materials

Fadraciclib (CYC065) was provided by Cyclacel Pharmaceuticals Inc. (Dundee, United Kingdom). Flavopiridol was obtained from the Drug Synthesis and Chemistry Branch, Division of Cancer Treatment, National Cancer Institute (Bethesda, MD). Roscovitine was purchased from LKT Laboratories, Inc (St. Paul, MN). SNS-032 was provided by Sunesis Pharmaceuticals Inc. (South San Francisco, CA). Venetoclax (ABT-199) was purchased from Xcess Biosciences (Chicago, IL). All of the above compounds were dissolved in dimethyl sulfoxide (DMSO) at 10 mM and stored at –80°C in small aliquots. Fludarabine (9-β-D-arabinofuranosyl-2-fluoroadenine or F-ara-A; Sigma-Aldrich), was prepared as a 2 mM stock solution in sterile water and stored at –20°C. Dactinomycin and cycloheximide were purchased from Sigma-Aldrich. [^3^H]uridine (50 Ci/mmol) was purchased from Moravek Biochemicals Inc. (Brea, CA). Annexin V–fluorescein isothiocyanate (FITC) and annexin V–Cy5 were purchased from BD Biosciences (Franklin Lakes, NJ). DiOC6(3) and propidium iodide (PI) solution (1 mg/ml) was purchased from Sigma-Aldrich. Z-VAD-FMK (methyl ester) was purchased from MP Biomedicals (Solon, OH). The human recombinant IL-4 was purchased from Sigma-Aldrich. Mouse monoclonal antibody against CD40 was purchased from GeneTex Inc. (Irvine, CA). The goat F(ABʹ)_2_ fragment to human IgM was purchased from MP Biomedicals or Jackson ImmunoResearch Inc. (West Grove, PA).

#### Isolation of CLL lymphocytes

Peripheral blood from the CLL patients and healthy donors were collected in heparin vacutainer tubes and centrifuged at 1500 rpm for 15 min to separate the plasma. The plasma (upper layer) was removed and saved for cell culture. The lower layer was diluted with phosphate-buffered saline, and the mononuclear cells were isolated by Ficoll density-gradient centrifugation. The isolated cells were cultured at 1 × 10^7^ cells/ml in RPMI 1640 medium containing 10% of autologous plasma. Both fresh and frozen CLL samples were used in this study. We observed similar drug responses in the fresh or frozen-thawed samples measured by both immunoblotting and flow cytometry.

**Quantitation of mitochondrial membrane potential loss and cell death by flow cytometry**

Change in mitochondrial membrane potential was measured using the fluorescent cation DiOC6(3) and PI. Cell death was evaluated with annexin V and PI. CLL cells (1×10^6^ cells) were incubated in the dark for 15 min at room temperature in 200 μl annexin binding buffer (BD Biosciences) with either 5 μl annexin V–FITC or annexin V–Cy5 plus 20 nM DiOC6(3). After staining, 300 μl of binding buffer with 5 μl of 50 μg/ml PI was added to each tube. Samples were run immediately with a Becton Dickinson FACSCalibur flow cytometer (Becton Dickinson, Franklin Lakes, NJ) and analyzed by FlowJo (Tree Star Inc., Ashland, OR), or with the Accuri C6 flow cytometer (Becton Dickinson). Cells with intact mitochondrial membrane potential were stained positive for DiOC6(3) but negative for PI. Cells stained positive for either annexin V or PI were considered dead. To access the viability of normal lymphocytes from healthy donors after incubation with fadraciclib, the samples were triple-stained with annexin V–FITC, mouse anti-human CD3-APC, and mouse anti-human CD19-RPE (Life Technologies). The apoptotic cells (annexin V–positive) were measured in subpopulations staining positive for CD3 (T cells) or CD19 (B cells).

#### Measuring RNA synthesis

RNA synthesis was measured by quantitating incorporation of [^3^H]uridine into the perchloric acid-insoluble materials. Briefly, after incubation with the drugs, CLL cells were labeled for 1 h with [^3^H]uridine (10 μCi/ml). The cells were then washed twice with 10 ml of ice-cold PBS and then lysed with 0.5 ml H_2_O and 0.5 ml of 0.8 N perchloric acid. Following centrifugation, the pellet was washed once with 1 ml of 0.4 N perchloric acid, then dissolved in 1 ml of H_2_O with 50 µl of 10 N KOH overnight. The supernatant was then transferred to scintillation vials to count radioactivity.

#### Immunoblotting

CLL cells were lysed in radioimmunoprecipitation assay buffer (Sigma-Aldrich) by sonication. Protein content in the lysate was determined with the Bio-Rad DC Protein Assay kit (Bio-Rad Laboratories, Hercules, CA). Cell lysate proteins (20 µg) were separated by sodium dodecyl sulfate–polyacrylamide gel electrophoresis and then electro-transferred to a nitrocellulose membrane (GE Osmonics Labstore, Minnetonka, MN). The membranes were blocked for 1 h in the blocking buffer (LI-COR Biosciences, Lincoln, NE) and then incubated with primary antibodies for 3 h, followed by secondary antibodies conjugated with fluorescent dyes for 1 h. The blots were scanned by an Odyssey Infrared Imaging system to obtain images and quantitations (LI-COR Biosciences). For Mcl-1 protein quantitation, we performed serial dilutions of pure Mcl-1 protein and confirmed that the Mcl-1 signal in our immunoblots increased in a linear fashion with the amount of Mcl-1 protein. The recombinant human Mcl-1 protein was purchased from Abcam (Cambridge, UK). Antibodies for total RNA Pol II (8WG16) and phosphorylated C-terminal domain at Ser2 (H5) or Ser5 (H14) were purchased from Covance Research Products, Inc. (Berkeley, CA). The antibodies to Mcl-1 (S-19) and Bcl-2 (100) were purchased from Santa Cruz Biotechnology (Dallas, TX). The antibody to PARP was from Biomol International Inc. (Plymouth Meeting, PA). The XIAP antibody was from BD Biosciences Pharmingen (San Diego, CA). The antibody to actin was from Sigma-Aldrich. The antibody for GAPDH was purchased from Cell Signaling Technology (Danvers, MA). Alexa Fluor 680 goat anti-mouse IgG and Alexa Fluor 680 goat anti-mouse IgM were purchased from Invitrogen (Carlsbad, California). IRDye 800CW goat anti-rabbit IgG and IRDye 800CW goat anti-mouse IgG were from LI-COR Biosciences.

**RNA isolation and real-time quantitative PCR**

Total cellular RNA was isolated from the primary CLL cells by QIAcube using the RNeasy Mini Kit (QIAGEN, Valencia, CA) with DNase digestion to completely remove the genomic DNA. Total RNA (20-50 ng) was used for the one-step real-time polymerase chain reaction (RT-PCR) in the TaqMan One-Step RT-PCR Master Mix (Applied Biosystems, Foster City, CA). Each PCR reaction was carried out in a 25-µl volume on a 96-well optical reaction plate for 30 min at 48°C for the reverse transcription reaction, followed by 10 min at 95°C for initial denaturing, then 40 cycles at 95°C for 15 sec and 60°C for 2 min in the 7900HT Sequence Detection System (Applied Biosystems). The relative gene expression was analyzed by the comparative Ct method using 18s ribosomal RNA as an endogenous control. All primers and probes and RT-PCR reaction buffers were purchased from Applied Biosystems.

**Evaluation of the combination effect**

The combination effect of fadraciclib and venetoclax was assessed by annexin V/PI assays after CLL cells had been incubated for 24 h with each individual drug and in combination. The combinations were carried out in a constant ratio based on the IC_50_ of each individual drug. The combination effects were evaluated using the CalcuSyn software (Biosoft, Cambridge, UK), which was developed based on the median-effect method created by Chou and Talalay^1^. A calculated combination index of <1 indicates synergy, 1 indicates additive, and >1 indicates antagonism.

#### Statistical analysis

Statistical analysis was carried out using the GraphPad Prism software (GraphPad Software, Inc., San Diego, CA). A p-value less than 0.05 was considered statistically significant.

#### References:

1. Chou TC, Talalay P. Quantitative analysis of dose-effect relationships: the combined effects of multiple drugs or enzyme inhibitors. Advances in enzyme regulation. 1984; 22:27-55.
